# Giant Abdominal Wall Endometrioma: A Case Report

**DOI:** 10.7759/cureus.12766

**Published:** 2021-01-18

**Authors:** Batool M Alsamahiji, Mohammed A Albaqshi, Areej J Alolayan, Hassan A Alzayer, Mohammad A Alalwan, Hind M Faqeeh, Fatimah A Al Zaher, Afnan M Maashi, Ammar A Aljeshi

**Affiliations:** 1 College of Medicine, King Fahd Hospital of the University, Al-Khobar, SAU; 2 College of Medicine, Medical University of Warsaw, Warsaw, POL; 3 College of Medicine, Jazan University, Jazan, SAU; 4 College of Medicine, Arabian Gulf University, Manama, BHR

**Keywords:** endometrioma, abdominal pain, cesarean section

## Abstract

Endometriosis is defined as the presence of endometrial glands and stroma outside the uterine cavity. Endometriosis may involve a wide spectrum of anatomic locations, but it typically involves pelvic locations. We report the case of a 45-year-old woman who presented with a history of abdominal pain and swelling. She first noticed the swelling eight months prior to presentation, and it had gradually progressed in size. The patient reported that the swelling increased in size during menses. Physical examination revealed a well-defined firm mass to the right of the midline. The mass had a smooth surface but limited mobility after abdominal wall muscle contraction, suggesting an infiltration of the underlying muscular structures. The findings demonstrated by computed tomography of the abdomen confirmed the diagnosis of abdominal wall endometrioma. The patient underwent successful resection of the lesion with complete resolution of her symptoms.

## Introduction

Endometriosis is defined as the presence of endometrial glands and stroma outside the uterine cavity. It represents a common gynecologic problem affecting around 10% of women globally [1]. It is estimated that endometriosis constitutes up to 70% of pelvic pain in women and adolescents. Several studies have shown that factors associated with increased risk of endometriosis include prolonged estrogen exposure, nulliparity, shorter menstrual cycles, heavy menstrual bleeding, and being underweight [2]. Endometriosis typically presents with dysmenorrhea, dyspareunia, infertility, and bladder or bowel disturbances [3]. Endometriosis may involve a wide spectrum of anatomic locations, but it typically involves pelvic locations [4]. Herein, we report the case of a giant abdominal wall endometrioma.

## Case presentation

We present the case of a 45-year-old woman who presented to the outpatient department with a history of right-sided abdominal pain and swelling. She first noticed the swelling eight months ago, and it had gradually progressed in size. The patient reported that the swelling increased in size during menses and was interfering with her daily activities. The associated pain was moderately improved after the use of non-steroidal anti-inflammatory drugs. However, there were no changes in the overlying skin and she reported no history of bowel or bladder symptoms.

The patient underwent a cesarean section two years before the presentation due to fetal distress. Otherwise, she did not have any remarkable past medical history. She was not taking any medications. Her menstrual periods were regular and were not associated with menorrhagia. She did not have any prior history of gynecological conditions.

Physical examination revealed a well-defined firm mass to the right of the midline. The mass had a smooth surface but limited mobility after abdominal wall muscle contraction, suggesting an infiltration of the underlying muscular structures. Basic hematological and biochemical laboratory investigations were within normal limits.

In light of the aforementioned clinical information, a contrast-enhanced abdominal computed tomography (CT) was planned for further characterization of the abdominal lesion. The CT scan demonstrated a large lobulated mass with heterogenous enhancement, measuring 17 × 15 × 10 cm and infiltrating the underlying muscular structure, confirming the diagnosis of abdominal wall endometrioma. The mass lesion was noted to displace adjacent structures.

**Figure 1:**
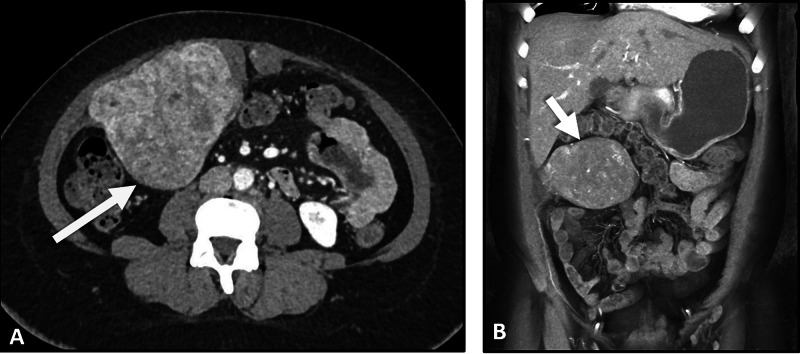
Axial (A) and coronal (B) abdominal CT images demonstrating a heterogenous left-sided abdominal mass lesion (arrow) with enhancement originating from the underlying muscular structures. CT, computed tomography

The patient underwent surgical resection of the abdominal wall endometrioma along with a part of the underlying rectus abdominis muscle. It was noted that the endometrioma had risen at the site of the previous scar of the cesarean section. There was minimal blood loss. The postoperative course was uneventful. The patient was discharged seven days after the operation. The patient was followed up in the outpatient department for six months and reported major satisfaction and complete resolution of her symptoms.

## Discussion

We reported the case of a giant abdominal wall endometrioma that underwent successful surgical management. Abdominal wall endometrioma typically develops after surgical procedures on the uterus, such as cesarean section. The development of endometriosis after surgical operation is most likely attributed to the spread of endometrial tissue into the surgical wound [5]. The estimated incidence of abdominal wall endometrioma after cesarean section is between 0.03% and 0.45% [3,6]. As the dissemination of endometrial tissue into the surgical scar is common after cesarean section, the rarity of abdominal wall endometrioma suggests that genetic factors could play a role. Additionally, some cases of spontaneous abdominal wall endometrioma in patients without a history of uterine surgeries have been reported.

The classic history of cyclic abdominal wall and swelling, as in the present case, is key in the diagnosis of abdominal wall endometrioma [7]. Ultrasound examination may aid in reaching the diagnosis by demonstrating a hypoechoic, vascular, and/or solid mass near the surgical incision [3]. However, the CT scan allows for more accurate evaluation and surgical planning. The size of abdominal wall endometrioma is highly variable. The present case demonstrated that endometrioma may present as a giant lesion causing a pressure effect on the underlying structures. The differential diagnoses of the abdominal wall lesion, in the present case, include a wide spectrum of conditions, such as sarcoma, desmoid tumor, hematoma, or metastasis. Wide local excision is the treatment of choice and results in complete resolution of symptoms [2]. However, the recurrence of the condition is a known complication [7]. Of note, several factors have been identified to increase the risk of recurrence, such as younger age and prior use of medical treatment of endometriosis [6]. However, it should be remembered that complete resection of the lesion is the mainstay to prevent recurrence.

## Conclusions

We reported the case of a large abdominal wall endometrioma. Although very rare, this clinical entity should be kept in mind when encountering a woman with cyclic abdominal pain and swelling. The size of endometrioma can be very large which should not make the physician overlook this condition. Surgical resection is the treatment of choice and results in complete resolution of the symptoms.
